# Explaining health behavior: a new model centered around health experience and its determinants

**DOI:** 10.3389/fpsyg.2025.1626812

**Published:** 2025-09-22

**Authors:** Damien S. E. Broekharst, Sjaak Bloem, Edward A. G. Groenland, Tessa S. Folkertsma, Jim Ingebretsen Carlson, Frans Folkvord, Claire Everitt, Aad R. Liefveld, Giuseppe Fico, Francisco Lupiáñez-Villanueva

**Affiliations:** ^1^Center for Marketing and Supply Chain Management, Nyenrode Business University, Breukelen, Netherlands; ^2^Department of Gastroenterology, Medical Center Leeuwarden, Leeuwarden, Netherlands; ^3^Department of Rheumatology, Medical Center Leeuwarden, Leeuwarden, Netherlands; ^4^Department of Dermatology, Medical Center Leeuwarden, Leeuwarden, Netherlands; ^5^PredictBy, Barcelona, Spain; ^6^Department of Information and Communication Science, Universitat Oberta de Catalunya, Barcelona, Spain; ^7^Department of Communication and Cognition, Tilburg University, Tilburg, Netherlands; ^8^Devices Centre of Excellence, Pfizer, Cambridge, United Kingdom; ^9^Link2Trials, Hilversum, Netherlands; ^10^Life Supporting Technologies Research Group, Universidad Politécnica de Madrid, Madrid, Spain

**Keywords:** health behavior, health perceptions, experienced health, projected health, adjustment, acceptance, control

## Abstract

**Introduction:**

Conventional health-related models used to predict health behaviors have limited predictive power, as they fail to accurately capture an individual’s health experience, which correlates more closely with health behavior. Therefore, some researchers have aimed to develop a predictive model focused on subjective health experience and its determinants. Although this model may be promising, it is still rudimentary. Hence, this study aimed to explore a new extended subjective health experience model and segment it along the lines of relevant demographic variables to further improve health behavior predictions.

**Method:**

An online questionnaire was administered to a panel of 2,550 Dutch citizens, covering sample characteristics and measuring health perceptions, acceptance, control, projected health, experienced health, adjustment, and health behavior. Data were analyzed using descriptive, reliability, validity, and model statistics.

**Result:**

The analysis revealed that almost all assumed direct relationships within the overall and segmented models are statistically significant, making them exceptionally robust. It also became clear that health perception indirectly influences health behavior through several pathways. The strongest indirect pathways linking health perception to health behavior involve sequential mediation by acceptance, experienced health, and projected health, with control potentially preceding or replacing acceptance. The most moderate indirect pathways involve acceptance with either experienced or projected health, with control potentially preceding or replacing acceptance. The weaker indirect pathways are those involving adjustment either combined with experienced and projected health or embedded within more extended sequences. It further became evident that the model explained between 39.2 and 50.9% of the variance in health behavior.

**Conclusion:**

Healthcare professionals and other stakeholders may benefit from using key concepts such as acceptance, control, experienced health, and projected health to guide the development and implementation of future behavioral interventions.

## 1 Introduction

Over the past few decades, researchers from various fields have attempted to make accurate predictions about health behavior, which refers to health-related practices that can damage or improve the overall health of an individual (Jakovljevic and Ogura, 2016). In some scientific fields (e.g., health economics), the rational choice approach has become the dominant paradigm for predicting health behavior, whereas other scientific fields (e.g., health psychology, public health) came to rely on the social cognitive approach (Campbell, 2013; Armitage and Conner, 2000; Norman and Conner, 2005). The rational choice approach to predicting health behavior is based on rational choice theory and other associated theories (e.g., theory of planned behavior, theory of reasoned action). It traditionally infers health behavior from an individual’s preferences and choices (Campbell, 2013). The social cognitive approach to predicting health behavior is based on social cognitive theory and other associated theories (e.g., the theory of self-efficacy and health belief model) and posits that health behavior can be predicted by deploying intentions, attitudes, and beliefs (Armitage and Conner, 2000; Norman and Conner, 2005). However, scientific evidence suggests that both preferences and choices, as well as intentions, attitudes, and beliefs, have only limited predictive power about health behavior (Conell-Price and Jamison, 2015; Calnan and Rutter, 1986; Trankle and Haw, 2009). This predictive power is limited due to the inability of these factors to effectively represent an individual’s health experience, which has been shown to correlate more closely with health behavior (Conell-Price and Jamison, 2015; Calnan and Rutter, 1986; Trankle and Haw, 2009).

Therefore, Bloem and Stalpers aimed to develop and validate a new predictive model in which the concept of subjective health experience and its determinants play a central role, also known as the subjective health experience model (Stalpers, 2009; Bloem and Stalpers, 2012; Bloem, 2008; Bloem et al., 2020). Subjectively experienced health is best understood as a specific type of health-related quality of life, which could be defined as the perceived impact of health and treatment on an individual’s physical, mental, and social functioning (Stalpers, 2009; Bloem and Stalpers, 2012; Bloem, 2008; Bloem et al., 2020). This, in turn, falls under the broader category of quality of life, which can be defined as the overall perception of one’s position in life based on personal goals, expectations, cultural context, and values (Stalpers, 2009; Bloem and Stalpers, 2012; Bloem, 2008; Bloem et al., 2020). Subjective health experience refers to an individual’s experience of physical and mental functioning while living their life according to their wishes, within the actual constraints and limitations of individual existence, and is influenced by two key factors, namely acceptance and control (Stalpers, 2009; Bloem and Stalpers, 2012; Bloem, 2008; Bloem et al., 2020). Acceptance expresses the extent to which individuals can experience their health condition as an integral part of their existence, while control expresses the extent to which individuals believe themselves to be able to exert influence over their condition (Bloem et al., 2020). The acceptance and control exhibited by an individual are, however, neither self-evident nor independent factors, and they ultimately depend on an individual’s health perception, which refers to the momentary sensing, understanding and interpreting of one’s quality of life, well-being, and overall health rooted in and emerging from one’s specific situational context (Henchoz et al., 2008). Although the subjective health experience model might be a promising alternative to traditional ways of predicting health behavior, it can still be considered rather rudimentary and simplistic due to three important shortcomings (Henchoz et al., 2008).

**Figure 1:**
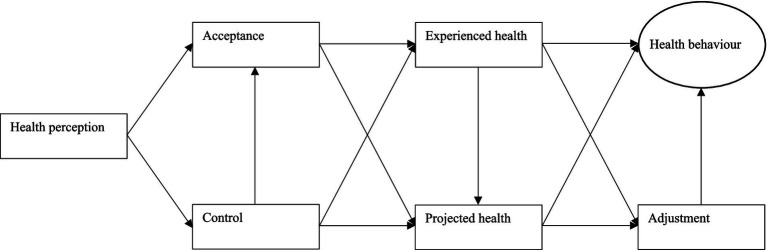
The new extended subjective health experience model.

First, the model only theoretically assumes that health experience affects health behavior without explicitly including the direct relationship between health experience and health behavior in the model. Second, the model also does not encompass the indirect relationship between health experience and health behavior through the process of adjustment, which refers to an individual’s ability to adapt to unexpected circumstances. Third, the model focuses solely on past health experience without considering future health projection, which entails the estimation or forecasting of future health states. To address these shortcomings, this study introduces and explores a new extended subjective health experience model that includes these missing components and improves predictions of health behavior (Figure 1). Additionally, this study also segments the new extended subjective health experience model based on important demographic variables, namely age, gender, region, education, and diagnosis, to generate more insight into the health behavior of particular subgroups. By examining the predictive power of this new extended model, it will become clear whether healthcare professionals and other stakeholders should consider deploying its key concepts, such as health perception, acceptance, control, and experienced or projected health, as starting points for the development and implementation of future behavioral interventions.

## 2 Methods

### 2.1 Design, procedure, and participants

To explore the new extended subjective health experience model, a quantitative research design was implemented using online questionnaires. These surveys were distributed to a substantial cohort of 2,550 Dutch citizens, organized by the IPSOS research agency. Panel members received invitations via email, along with comprehensive information and a formal consent request. This study only included those who provided consent for their responses to be used in future research. Data for this investigation were collected from September to December 2021. The questionnaires administered to panel members encompassed various items based on sample characteristics, followed by multiple measurement instruments covering health perceptions, acceptance, control, projected health, experienced health, adjustment, and health behavior.

### 2.2 Questionnaire, items, and scales

Sample characteristics such as age, gender, region, education, and diagnosis were delineated using individual items. These items were assessed on nominal scales employing dichotomous response categories or ordinal scales with ascending response categories. The perception of quality of life, well-being, and overall health was evaluated through the EuroQol Five-Dimensions Five-Level questionnaire (EQ-5D-5L), encompassing dimensions like mobility, self-care, usual activities, pain and discomfort, as well as anxiety and depression (Thompson and Turner, 2020; Devlin and Brooks, 2017; Brazier et al., 2019). These dimensions were measured on a 5-point scale (Thompson and Turner, 2020; Devlin and Brooks, 2017; Brazier et al., 2019). Acceptance, control, and adjustment were assessed using their respective scales from the Bloem & Stalpers questionnaire (Stalpers, 2009; Bloem and Stalpers, 2012; Bloem, 2008; Bloem et al., 2020). Each concept was measured with three items using a 7-point Likert scale ranging from 1 = fully disagree to 7 = fully agree (Stalpers, 2009; Bloem and Stalpers, 2012; Bloem, 2008; Bloem et al., 2020). Experienced and projected health were assessed using the Bloem & Stalpers ladders, measuring physical health, psychological health, social health, and general health for the previous or subsequent month (Stalpers, 2009; Bloem and Stalpers, 2012; Bloem, 2008; Bloem et al., 2020). Scores were determined on an 11-level scale in which level 1 represented the worst day and level 11 the best day (Stalpers, 2009; Bloem and Stalpers, 2012; Bloem, 2008; Bloem et al., 2020). Health behavior was appraised using an instrument based on the BRAVO dimensions covering exercise, nutrition, rest, smoking, alcohol use, and general health (Peeters et al., 2015). Responses for these dimensions were recorded on a 6-point Likert scale ranging from 1 = fully disagree to 6 = fully agree (Peeters et al., 2015).

### 2.3 Analysis, interpretation, and software

Sample characteristics were defined using descriptive statistics. Continuous variables were presented as means, while categorical variables were presented as percentages. Questionnaire characteristics were analyzed through analyzes of construct reliability, construct validity, convergent validity, and discriminant validity statistics. Construct reliability was evaluated using coefficients such as Cronbach’s alpha (α), rho_a, and rho_c, with values indicating reliability when exceeding the 0.70 threshold (Hair et al., 2019). Construct validity was established through factor analysis, confirming and optimizing the factorial structure of measurement instruments by removing items with multiple loadings (Hair et al., 2019). Convergent validity was assessed by examining the average variance extracted (AVE) coefficient, which is considered sufficient if surpassing the 0.50 threshold (Hair et al., 2019). Discriminant validity was scrutinized using the heterotrait-monotrait (HTMT) ratio, which was deemed adequate if below the 0.90 threshold (Hair et al., 2019). Model characteristics were assessed through partial least squares structural equation modeling (PLS-SEM), with results reported via effect sizes, significance levels, and explained variance. The effect sizes, indicated by standardized beta-coefficients (β), are considered small below 0.25, medium between 0.25 and 0.50, and large above 0.50 (Hair et al., 2019). Significance levels, determined by *p*-values, are considered significant if below the 0.05 threshold (Hair et al., 2019). The explained variance, measured by R-squared (R^2^) coefficients, is considered weak below 0.25, moderate between 0.25 and 0.50, and strong above 0.50 (Hair et al., 2019). Sample characteristics were obtained using IBM SPSS Statistics Version 27, and analyzes of questionnaire and model characteristics were conducted using SmartPLS Version 4.0 (Hair et al., 2019; Bala, 2016; Dudley et al., 2004).

## 3 Results

### 3.1 Sample characteristics

The study analyzed a final sample of 2,550 panel members, which closely mirrors the Dutch population in terms of age (CBS, 2024a), gender (RIVM, 2024a), region (PBL, 2024; CBS, 2024b), education (CBS, 2024c), and diagnosis distribution (RIVM, 2024b). However, the final sample is skewed toward a more urbanized population (PBL, 2024; RIVM, 2024b). In line with Dutch guidelines and other studies, participants aged 60 years and above are considered older adults (Broekharst et al., 2023). Detailed sample characteristics are provided in Table 1.

**Table 1:** Sample description.

Variables				
Age	68.7% < 60 years	31.3% > 60 years		
Gender	48.6% male	51.4% female		
Region	56.8% city	34.3% suburb	8.9% rural	
Education	31.5% lower	29.3% average	38.9% higher	0.4% unknown
Diagnosis	37.2% healthy	27.9% 1 disease	34.9% comorbidities	

### 3.2 Instrument characteristics

The instruments on health perceptions (α = 0.806; rho_a = 0.824; rho_c = 0.866), acceptance (α = 0.885; rho_a = 0.892; rho_c = 0.928), control (α = 0.873; rho_a = 0.878; rho_c = 0.922), adjustment (α = 0.797; rho_a = 0.810; rho_c = 0.880), experienced health (α = 0.857; rho_a = 0.861; rho_c = 0.903), projected health (α = 0.881; rho_a = 0.887; rho_c = 0.918), and health behavior (α = 0.721; rho_a = 0.766; rho_c = 0.823) demonstrated reliability with coefficients exceeding 0.70. The instruments on health perceptions, acceptance, control, adjustment, experienced health, and projected health had sufficient construct validity as all their items loaded on a single component, while the instrument on health behavior also had sufficient construct validity after excluding two double-loading items (i.e., alcohol use and smoking). The instruments on health perceptions (AVE = 0.566), acceptance (AVE = 0.812), control (AVE = 0.796), adjustment (AVE = 0.709), experienced health (AVE = 0.700), projected health (AVE = 0.737), and health behavior (AVE = 0.540) demonstrated sufficient convergent validity with coefficients exceeding 0.50. The instruments on health perceptions (HTMT = 0.571–0.669), acceptance (HTMT = 0.629–0.822), control (HTMT = 0.571–0.766), adjustment (HTMT = 0.579–0.822), experienced health (HTMT = 0.602–0.868), projected health (HTMT = 0.605–0.868), and health behavior (HTMT = 0.581–0.732) demonstrated sufficient discriminant validity as coefficients remained below 0.90.

### 3.3 Model characteristics

The new extended subjective health experience model will be explained through the presentation of direct and indirect relationships as well as the explained variance across the overall model and its age-segmented, gender-segmented, region-segmented, education-segmented, and diagnosis-segmented variants. It should be mentioned that the theoretically assumed relationships in each of these models were well supported by empirical evidence, whereas this was not the case when these relationships were inverted, decreasing the probability of reverse causality.

#### 3.3.1 Direct relationships

From the analysis of model characteristics (see Table 2), it stems that health perception has a modest to moderate statistically significant positive relationship with acceptance (β = 0.229–0.361) and a moderate to large statistically significant positive relationship with control (β = 0.370–0.538) across the overall and segmented models. Control demonstrates a moderate to large statistically significant positive relationship with acceptance (β = 0.398–0.577) and a modest to moderate statistically significant positive relationship with experienced health (β = 0.068–0.264) and projected health (β = 0.084–0.213) across the overall and segmented models, except for the non-significant relationship between control and experienced health among rural individuals. Acceptance has a moderate to large statistically significant positive relationship with experienced health (β = 0.466–0.583) and a modest statistically significant positive relationship with projected health (β = 0.124–0.208) across the overall and segmented models. Experienced health has a large statistically significant positive relationship with projected health (β = 0.495–0.625), a modest statistically significant positive relationship with health behavior (β = 0.097–0.190), and a moderate statistically significant positive relationship with adjustment (β = 0.288–0.393) across the overall and segmented models, except for the non-significant relationship between experienced health and health behavior among rural individuals. Projected health has a moderate statistically significant positive relationship with health behavior (β = 0.324–0.418) and a modest to moderate statistically significant positive relationship with adjustment (β = 0.205–0.317) across the overall and segmented models. Finally, adjustment has a modest to moderate statistically significant positive relationship with health behavior (β = 0.209–0.340) across the overall and segmented models.

**Table 2:** Model characteristics.

	Age (β)		Gender (β)		Region (β)			Education (β)			Diagnosis (β)			Overall (β)
	<60	>60	Male	Female	City	Suburbs	Rural	Lower	Average	Higher	Without	One	Multiple	
Direct relationships														
Health perception ➔ Acceptance	0.263*	0.331*	0.264*	0.294*	0.281*	0.264*	0.361*	0.292*	0.269*	0.277*	0.229*	0.283*	0.322*	0.282*
Health perception ➔ Control	0.480*	0.523*	0.455*	0.519*	0.459*	0.542*	0.501*	0.538*	0.502*	0.436*	0.370*	0.431*	0.507*	0.493*
Control ➔ Acceptance	0.537*	0.539*	0.540*	0.536*	0.564*	0.543*	0.398*	0.567*	0.551*	0.508*	0.577*	0.549*	0.477*	0.540*
Acceptance ➔ Experienced health	0.509*	0.501*	0.488*	0.544*	0.545*	0.466*	0.583*	0.557*	0.508*	0.514*	0.513*	0.477*	0.543*	0.522*
Acceptance ➔ Projected health	0.162*	0.141**	0.129*	0.182*	0.131*	0.189*	0.199**	0.156**	0.171*	0.138*	0.147*	0.208*	0.124**	0.157*
Control ➔ Experienced health	0.157*	0.228*	0.191*	0.150*	0.130*	0.264*	0.068	0.152*	0.179*	0.168*	0.114**	0.215*	0.162*	0.170*
Control ➔ Projected health	0.138*	0.121*	0.116*	0.150*	0.113*	0.151*	0.213*	0.122*	0.167*	0.119*	0.094*	0.084**	0.199*	0.134*
Experienced health ➔ Projected health	0.566*	0.625*	0.606*	0.562*	0.620*	0.546*	0.495*	0.604*	0.543*	0.603*	0.599*	0.562*	0.582*	0.584*
Experienced health ➔ Health behavior	0.125*	0.190*	0.154*	0.142*	0.148*	0.170*	0.097	0.142**	0.160*	0.139**	0.143*	0.136**	0.139**	0.147*
Experienced health ➔ Adjustment	0.288*	0.375*	0.349*	0.303*	0.288*	0.393*	0.291*	0.323*	0.293*	0.357*	0.332*	0.296*	0.321*	0.324*
Projected health ➔ Health behavior	0.366*	0.362*	0.358*	0.369*	0.325*	0.418*	0.377*	0.366*	0.384*	0.351*	0.350*	0.324*	0.396*	0.364*
Projected health ➔ Adjustment	0.283*	0.245*	0.222*	0.317*	0.289*	0.227*	0.356*	0.309*	0.307*	0.205*	0.228*	0.312*	0.284*	0.275*
Adjustment ➔ Health behavior	0.254*	0.267*	0.271*	0.264*	0.285*	0.216*	0.340*	0.287*	0.266*	0.245*	0.270*	0.337*	0.209*	0.266*
Indirect relationships														
Health perceptions ➔ Health behavior	0.218*	0.301*	0.218*	0.268*	0.221*	0.293*	0.268*	0.283*	0.263*	0.203*	0.170*	0.225*	0.261*	0.247*
Health perception ➔ Acceptance ➔ Projected health ➔ Health behavior	0.016*	0.017**	0.012*	0.020*	0.012*	0.021**	0.027	0.017**	0.018**	0.013**	0.012**	0.019*	0.016**	0.016*
Health perception ➔ Control ➔ Projected health ➔ Health behavior	0.024*	0.023**	0.019*	0.029*	0.017*	0.034*	0.040 **	0.024**	0.032*	0.018**	0.012**	0.012**	0.040*	0.024*
Health perception ➔ Control ➔ Acceptance ➔ Projected health ➔ Adjustment ➔ Health behavior	0.003*	0.003	0.002**	0.004*	0.003**	0.003**	0.005	0.004**	0.004**	0.002**	0.002**	0.005**	0.002	0.003*
Health perception ➔ Control ➔ Experienced health ➔ Projected health ➔ Health behavior	0.016*	0.027*	0.019*	0.016*	0.012*	0.033*	0.006	0.018**	0.019*	0.016*	0.009**	0.017*	0.019*	0.018*
Health perception ➔ Control ➔ Experienced health ➔ Projected health ➔ Adjustment ➔ Health behavior	0.003*	0.005**	0.003*	0.004*	0.003*	0.004**	0.002	0.004**	0.004**	0.002**	0.002**	0.005*	0.003**	0.004*
Health perception ➔ Acceptance ➔ Experienced health ➔ Health behavior	0.017*	0.031**	0.020*	0.023*	0.023*	0.021**	0.020	0.023**	0.022**	0.020**	0.017**	0.018**	0.024**	0.022*
Health perception ➔ Control ➔ Experienced health ➔ Health behavior	0.009**	0.023**	0.013**	0.011**	0.009**	0.024**	0.003	0.012**	0.014**	0.010**	0.006**	0.013**	0.011**	0.012*
Health perception ➔ Control ➔ Experienced health ➔ Adjustment ➔ Health behavior	0.006*	0.012**	0.008*	0.006*	0.005*	0.012*	0.003	0.008**	0.007**	0.006*	0.004**	0.009**	0.005**	0.007*
Health perception ➔ Acceptance ➔ Projected health ➔ Adjustment ➔ Health behavior	0.003*	0.003	0.002**	0.004*	0.003**	0.002**	0.009	0.004**	0.004**	0.002**	0.002**	0.006**	0.002	0.003*
Health perception ➔ Control ➔ Acceptance ➔ Experienced health ➔ Health behavior	0.016*	0.027**	0.018*	0.022*	0.021*	0.023*	0.011	0.024**	0.022**	0.016**	0.016**	0.015**	0.018**	0.020*
Health perception ➔ Acceptance ➔ Experienced health ➔ Projected health ➔ Adjustment ➔ Health behavior	0.005*	0.007*	0.005*	0.008*	0.008*	0.003**	0.013**	0.009*	0.006**	0.004*	0.004*	0.008*	0.006*	0.006*
Health perception ➔ Control ➔ Acceptance ➔ Experienced health ➔ Adjustment ➔ Health behavior	0.010*	0.014*	0.011*	0.012*	0.012*	0.012*	0.011**	0.016*	0.011*	0.010*	0.010*	0.011*	0.009*	0.012*
Health perception ➔ Control ➔ Acceptance ➔ Experienced health ➔ Projected health ➔ Health behavior	0.027*	0.032*	0.026*	0.031*	0.028*	0.031*	0.022**	0.038*	0.029*	0.024*	0.023*	0.021*	0.030*	0.030*
Health perception ➔ Control ➔ Acceptance ➔ Projected health ➔ Health behavior	0.015*	0.014**	0.011*	0.019*	0.011*	0.023*	0.015**	0.017**	0.018*	0.011*	0.011*	0.016*	0.012**	0.015*
Health perception ➔ Acceptance ➔ Experienced health ➔ Projected health ➔ Health behavior	0.028*	0.037*	0.028*	0.033*	0.031*	0.028*	0.039**	0.036*	0.028*	0.030*	0.025*	0.025*	0.040*	0.031*
Health perception ➔ Control ➔ Acceptance ➔ Experienced health ➔ Projected health ➔ Adjustment ➔ Health behavior	0.005*	0.006*	0.004*	0.007*	0.007*	0.004*	0.007**	0.009*	0.006*	0.003*	0.004*	0.007*	0.005*	0.006*
Health perception ➔ Acceptance ➔ Experienced health ➔ Adjustment ➔ Health behavior	0.010*	0.017*	0.012*	0.013*	0.013*	0.010*	0.021**	0.015*	0.011*	0.012*	0.011*	0.014*	0.012*	0.013*
Health perception ➔ Control ➔ Projected health ➔ Adjustment ➔ Health behavior	0.005*	0.004**	0.003**	0.007*	0.004**	0.004**	0.013**	0.006**	0.007**	0.003**	0.002**	0.004	0.006*	0.005*
Explained variance	40.4%	50.9%	44.3%	45.3%	41.9%	49.5%	49.8%	48.1%	48.6%	39.2%	41.6%	46.6%	42.3%	44.8%

*p ≤ 0.001.

**p ≤ 0.05.

#### 3.3.2 Indirect relationships

The analysis of model characteristics (see Table 2) indicates that health perception has a modest to moderately statistically significant indirect positive relationship with health behavior (β = 0.170–0.301) across the overall and segmented models. This total indirect relationship constitutes a cumulative association derived from the aggregation of all intermediary pathways linking health perception to health behavior, each of which is typically modest in magnitude but often statistically significant. The indirect pathway from perception to health behavior through control and experienced health is modest but statistically significant (β = 0.003–0.024) across models, except for that of rural individuals. The indirect pathway between perception and health behavior through acceptance and experienced health is minor but statistically significant (β = 0.017–0.031) across models, except for that of rural individuals. The indirect pathway from perception to health behavior through control and projected health is small but statistically significant (β = 0.012–0.040) across models. The indirect pathway between perception and health behavior via acceptance and projected health is modest but statistically significant (β = 0.012–0.027) across models, except for that of rural individuals. The indirect pathway from perception to health behavior through control, experienced health, and projected health is minor but statistically significant (β = 0.006–0.033) across models, except for that of rural individuals. The indirect pathway between perception and health behavior via acceptance, experienced health, and projected health is small but statistically significant (β = 0.025–0.040) across models. The indirect pathway from perception to health behavior through control, experienced health, and adjustment is modest but statistically significant (β = 0.003–0.012) across models, except for that of rural individuals. The indirect pathway between perception and health behavior via acceptance, experienced health, and adjustment is minor but statistically significant (β = 0.010–0.021) across models. The indirect pathway from perception to health behavior through control, projected health, and adjustment is small but statistically significant (β = 0.002–0.013) across models, except for that of individuals with one diagnosis. The indirect pathway between perception and health behavior via acceptance, projected health, and adjustment is modest but statistically significant (β = 0.002–0.009) across models, except for that of individuals over 60, rural individuals, and those with multiple diagnoses. The indirect pathway from perception to health behavior through control, experienced health, projected health, and adjustment is minor but statistically significant (β = 0.002–0.005) across models, except for that of rural individuals. The indirect pathway between perception and health behavior via acceptance, experienced health, projected health, and adjustment is small but statistically significant (β = 0.003–0.013) across models. The indirect pathway from perception to health behavior through control, acceptance, and experienced health is modest but statistically significant (β = 0.011–0.027) across models, except for that of rural individuals. The indirect pathway between perception and health behavior via control, acceptance, and projected health is minor but statistically significant (β = 0.011–0.023) across models. The indirect pathway from perception to health behavior through control, acceptance, experienced health, and projected health is small but statistically significant (β = 0.021–0.038) across models. The indirect pathway between perception and health behavior via control, acceptance, experienced health, and adjustment is modest but statistically significant (β = 0.009–0.016) across models. The indirect pathway from perception to health behavior through control, acceptance, projected health, and adjustment is minor but statistically significant (β = 0.002–0.005) across models, except for that of individuals over 60, rural individuals, and those with multiple diagnoses. The indirect pathway between perception and health behavior via control, acceptance, experienced health, projected health, and adjustment is small but statistically significant (β = 0.003–0.009) across models.

#### 3.3.3 Explained variance

The analysis of model characteristics (see Table 2) indicates that the overall and segmented models explain between 39.2 and 50.9% of the variance in health behavior. The overall model accounts for 44.8% of the variance in health behavior. When segmented by age, this model explains 40.4% of the variance among younger individuals and a higher 50.9% among older individuals. In terms of gender, this model explains 44.3% of the variance in male health behavior and 45.3% in female health behavior. When grouped by region, this model accounts for 41.9% of the variance among individuals living in cities, 49.5% of the variance among individuals living in suburbs, and 49.8% of the variance among individuals living in rural areas. When segmented by education level, this model explains 48.1% of the variance for those with lower education, 48.6% for those with average education, and a comparatively lower 39.2% for those with higher education. Finally, when grouped by diagnosis, this model explains 41.6% of the variance in individuals without a diagnosis, 46.6% in those with one diagnosis, and 42.3% in those with multiple diagnoses.

## 4 Discussion

In this study, a new extended subjective health experience model was explored and segmented along the lines of important demographic variables, namely age, gender, region, education, and diagnosis, to generate more comprehensive insight into the health behavior of particular subgroups. After analyzing the new extended subjective health experience model, three key findings became apparent.

The first key finding is that nearly all direct relationships within this new extended subjective health experience model are significant, making the overall and segmented models exceptionally robust. Health perception shows a strong relationship with acceptance and control. This seems logical, as how one perceives their health can either empower or hinder their ability to accept and manage health constraints (Eklund and Bäckström, 2006; Kim et al., 2016). Control shows a strong relationship with acceptance. This also seems explainable, as the control one exerts over their condition often facilitates its integration into their identity (Eklund and Bäckström, 2006; Kim et al., 2016). Acceptance has a strong relationship with experienced health and, to a lesser extent, projected health. This explanation seems reasonable, as different levels of acceptance could foster habituation or defiance, influencing the way health states are memorized or anticipated (Lee, 2014). Control has a modest relationship with experienced and projected health. This hypothesis seems valid, as different levels of control could foster self-efficacy or dependence, impacting the way health states are memorized or anticipated (Wallston, 1997). Experienced health shows a strong relationship with projected health. This phenomenon might be explained by the tendency to establish estimations of future health states based on memories of past health states, as these might provide a useful heuristic (Deco and Rolls, 2005). Experienced health suggests a modest relationship with health behavior and a moderate relationship with adjustment, leading to a moderate relationship with health behavior. This seems logical, as health experiences may foster learning processes and subsequent adjustment that could influence health behavior (Okano et al., 2000). Projected health suggests a moderate relationship with health behavior and adjustment, which also shows a moderate relationship with health behavior. This hypothesis seems self-evident, as estimations of future health states may activate approach or avoidance tendencies and subsequent adjustment processes leading to modified health behavior (De Ridder et al., 2008).

The second key finding is that, within this new extended subjective health experience model, health perception has a rather noticeable indirect relationship with health behavior, representing a cumulative impact of at least 18 typically modest but often significant intermediary pathways. The strongest indirect pathways linking health perception to health behavior involve sequential mediation by acceptance, experienced health, and projected health, with control potentially preceding or replacing acceptance. This relationship appears logical, as one’s perception of health is very likely to inform their sense of control and acceptance, which in turn shapes health experiences and the health projections arising from them, ultimately prompting specific health behaviors (Eklund and Bäckström, 2006; Kim et al., 2016; Lee, 2014; Wallston, 1997; Okano et al., 2000; De Ridder et al., 2008). The most moderate indirect pathways involve acceptance with either experienced or projected health, with control potentially preceding or replacing acceptance. This too appears logical, as it draws on the same variables and adheres to the same underlying rationale, yet the absence of a combined influence from experienced and projected health likely weakens the strength of these pathways (Eklund and Bäckström, 2006; Kim et al., 2016; Lee, 2014; Wallston, 1997; Okano et al., 2000; De Ridder et al., 2008). The weaker indirect pathways are those involving adjustment either combined with experienced and projected health or embedded within more extended sequences. This distinction may be attributable to the fact that adjustment itself constitutes a form of behavior, akin to health behavior, and may therefore absorb or diffuse part of the overall impact of these particular indirect pathways (Okano et al., 2000; De Ridder et al., 2008).

The third key finding is that this new extended subjective health experience model has considerable explanatory and predictive power regarding health behavior relative to existing models based on health intentions, attitudes, and beliefs central to the social cognitive approach, as well as the preferences and choices central to the rational choice approach. Research shows that health intentions, attitudes, and beliefs do not explain more than 28.0% of variance in health behavior and that health preferences and choices do not explain more than 30.7% of variance in health behavior, while the new extended subjective health experience model presented in this study explains between 39.2 and 50.9% of variance in health behavior (Conell-Price and Jamison, 2015; Calnan and Rutter, 1986; Trankle and Haw, 2009). It should be noted that the explained variance in health behavior is particularly high in older individuals, which might be explained by their relatively extensive experience with and habituation to suboptimal health states that inform their current health behavior (Schüz et al., 2014). Therefore, it may be especially worthwhile to deploy the key concepts of the alternative health behavior, such as acceptance, control, and the resulting subjective health experience, in the development and implementation of behavioral interventions for older individuals.

### 4.1 Strengths and limitations

This study exhibits various strengths and limitations. This study stands out for its considerable sample size, which significantly improves the generalizability and representativeness of its findings. Another strength of this study pertains to the considerable reliability and validity of the measurement instruments used, which enhances the veracity and accuracy of the findings. A possible limitation of this study pertains to its exclusively Dutch population and context, as well as its slight bias toward relatively urbanized respondents, which may distort and misrepresent findings. Another limitation of this study pertains to its cross-sectional nature, as this type of research does not account for potential changes in the measured relationships between variables in the overall and segmented models over time. A final limitation of this study concerns the timing of data collection during the COVID-19 period, which may have introduced potential distortion in participants’ perceptions of health and influenced the overall findings.

### 4.2 Practical implications

The findings of this study have certain implications for practice. The findings of this study imply that it might be beneficial to develop and implement health behavior interventions by influencing and nudging the experienced and projected health of individuals. The results of this study also imply that one can achieve this aim by specifically targeting the degree of acceptance and control perceived by individuals. The outcomes of this study further imply that in circumstances where individuals are unable to modify their sense of acceptance or control, it would be prudent to focus on recalibrating their perception of health itself. A notable example of the practical application of these findings is evident in the BEAMER project, where care pathways are thoughtfully tailored to enhance treatment adherence behaviors among patients across six countries and a range of disease contexts based on their subjective health experience and the underlying degree of control and acceptance (BEAMER, 2021).

### 4.3 Future research

Future research may pursue at least four main avenues. The first avenue for future research emphasizes the further segmentation of this model into other important demographic variables, such as annual income, ethnic background, and character traits. The second avenue for future research revolves around the validation of this model in different patient populations to better understand their disease-specific health behavior. The third avenue for future research highlights the validation of this model in various countries to understand the impact of geographical location and different cultures. The fourth avenue for future research focuses on co-creating and evaluating health behavior interventions based on the model presented in this study.

## 5 Conclusion

In this study, a new extended subjective health experience model was explored and segmented based on key sample characteristics, namely age, gender, residential region, education level, and diagnosis. After analysis, it became apparent that the core finding of this study is that nearly all assumed direct relationships within the overall model, as well as the segmented models, are statistically significant, making these models exceptionally robust. It also became clear that health perception indirectly affects health behavior through several pathways, of which those involving sequential mediation by acceptance, experienced health, and projected health, with control possibly preceding or replacing acceptance, seem to be the strongest. It further became evident that this new extended subjective health experience model explained more variance in health behavior than models based on health intentions, attitudes, and beliefs, as postulated in the social cognitive approach, or preferences and choices, as proposed in the rational choice approach. Therefore, it may be worthwhile for healthcare professionals and other stakeholders to consider deploying their key concepts, such as health perception, acceptance, control, and experienced or projected health, as starting points for the development and implementation of future behavioral interventions.

## Data Availability

The raw data supporting the conclusions of this article will be made available by the authors, without undue reservation.
